# Susceptibility to Superhelically Driven DNA Duplex Destabilization: A Highly Conserved Property of Yeast Replication Origins

**DOI:** 10.1371/journal.pcbi.0010007

**Published:** 2005-06-24

**Authors:** Prashanth Ak, Craig J Benham

**Affiliations:** UC Davis Genome Center, University of California, Davis, California, United States of America; University of California at San Diego, United States of America

## Abstract

Strand separation is obligatory for several DNA functions, including replication. However, local DNA properties such as A+T content or thermodynamic stability alone do not determine the susceptibility to this transition in vivo*.* Rather, superhelical stresses provide long-range coupling among the transition behaviors of all base pairs within a topologically constrained domain. We have developed methods to analyze superhelically induced duplex destabilization (SIDD) in genomic DNA that take into account both this long-range stress-induced coupling and sequence-dependent local thermodynamic stability. Here we apply this approach to examine the SIDD properties of 39 experimentally well-characterized autonomously replicating DNA sequences (ARS elements), which function as replication origins in the yeast *Saccharomyces cerevisiae*. We find that these ARS elements have a strikingly increased susceptibility to SIDD relative to their surrounding sequences. On average, these ARS elements require 4.78 kcal/mol less free energy to separate than do their immediately surrounding sequences, making them more than 2,000 times easier to open. Statistical analysis shows that the probability of this strong an association between SIDD sites and ARS elements arising by chance is approximately 4 × 10^−10^. This local enhancement of the propensity to separate to single strands under superhelical stress has obvious implications for origin function. SIDD properties also could be used, in conjunction with other known origin attributes, to identify putative replication origins in yeast, and possibly in other metazoan genomes.

## Synopsis

Several DNA functions require the two strands of the DNA duplex to transiently separate. Examples include the initiation of gene expression and of DNA replication. Here the authors examine the strand separation properties of the DNA duplex at autonomously replicating sequences (ARS elements), which are the potential replication origins in yeast.

In vivo, susceptibility to strand separation does not depend only on local DNA properties such as adenine plus thymine content or thermodynamic stability. Rather, stresses imposed on the DNA in vivo couple together the strand-opening behaviors of all base pairs that experience them. The authors use computational methods for analyzing stress-driven strand separation to examine the susceptibility to opening of 39 experimentally well-characterized ARS elements. They show that these ARS elements have strikingly increased susceptibilities to stress-induced separation relative to the surrounding sequences. On average, these ARS elements require 4.78 kcal/mol less free energy to separate than do surrounding sequences, making them more than 2,000 times easier to open. This enhanced susceptibility to stress-driven strand separation has obvious implications for the mechanisms that begin the process of replication. This property is also shared by bacterial and viral replication start points, suggesting that it may be a general attribute of replication origins.

## Introduction

In eukaryotes, DNA replication is initiated at multiple origins. Potential sites in the genome of the yeast *Saccharomyces cerevisiae* that may serve this function are referred to as autonomously replicating sequences, or ARS elements [1]. ARS elements are more A+T-rich than the genomic average, and contain regions of low local thermodynamic stability that are thought to be necessary for function [2,3]. However, the duplex unwinding required for replication initiation occurs as an isothermal process within topologically constrained domains of DNA. Under these conditions susceptibility to strand opening is not dependent only on local thermodynamic stability. Instead, superhelical stresses couple together the strand-opening behaviors of all base pairs that experience them. We hypothesize that the superhelical stresses that occur in vivo play a role in regulating the strand opening needed to initiate replication. This suggests that ARS elements should have an increased local susceptibility to superhelically induced duplex destabilization (SIDD). Here we demonstrate that virtually all known ARS elements do indeed show a significant local increase in their predicted SIDD susceptibility. Experiments on four specific ARS-containing regions have shown that each does experience local denaturation when negatively supercoiled [4].

We have calculated the SIDD properties of the entire yeast genome using a previously developed statistical mechanical method that includes both sequence-specific thermal stability and the global coupling induced by superhelical stresses within topological domains. (The algorithms implementing this method have been presented elsewhere [5].) This method computes the destabilization energy *G*(*x*) for each base pair (sometimes also called the SIDD energy) under the specified environmental conditions and level of superhelicity. This is the incremental free energy that is needed to guarantee separation of base pair *x* under these conditions. We note that *G*(*x*) is directly related to stability, not to destabilization—the higher the value of *G*(*x*), the more energy is needed to force that base pair open, and hence the more stable it is. Base pairs having *G*(*x*) near 10 kcal/mol remain essentially as stable under the assumed level of superhelicity as they would be in a relaxed molecule. (The majority of the base pairs act this way; significant superhelical destabilization is limited to a small fraction of the genome, as shown below.) Sites with *G*(*x*) near zero are strongly destabilized, and would denature with high probability under these conditions, while partially destabilized sites have intermediate values of *G*(*x*).

The SIDD energy *G*(*x*) is more informative regarding the extent of destabilization than is the probability *p*(*x*) of denaturation, because it also finds positions of partial destabilization. These can be biologically important because partial destabilization, even by only a few kilocalories, can greatly facilitate opening by other processes, such as interactions with regulatory molecules. For example, superhelical destabilization by only 3 kcal/mol (i.e., *G*(*x*) changing from 10 kcal/mol in a relaxed molecule to 7 kcal/mol under superhelicity), which is far less than is needed to open the duplex, will still increase the ease of opening by other processes by a factor of 130 (see Materials and Methods.) In this way changes in the level of imposed superhelicity can have strong effects on the rates of occurrence of regulatory events, especially those whose rate-limiting steps involve DNA strand opening.

Although these calculations have no free parameters, comparisons with experiments have shown that their predictions are quantitatively accurate. They determine the locations of opening and the extents of opening, both as functions of imposed superhelicity, at an accuracy comparable to experimental measurements in all sequences on which such experiments have been performed [6,7]. Many sites that these methods had previously calculated would open under stress have subsequently been experimentally shown to separate under these conditions, both in vitro and in vivo [8–11]. This gives confidence in the accuracy of their predictions when applied to other sequences on which experiments have not been performed.

**Figure 1:**
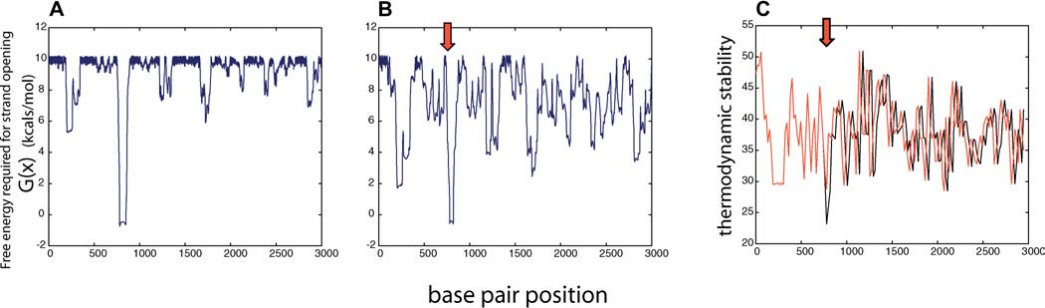
Comparison of Stress-Induced Duplex Destabilization Calculations with Assessments of Thermodynamic Stability (A) The SIDD profile showing the *G*(*x*) values computed for a 3-kbp region (525701–528700) on Chromosome X of yeast at superhelix density σ = −0.055. (B) The SIDD profile of a 38-bp deletion mutant of the same region [28], at the same superhelicity. The deletion is at positions 526489–526526, indicated by the red arrow. This deletion causes drastic changes of SIDD properties throughout the region, even 2 kbp away. This is an effect of the global coupling induced by the superhelical stresses. (C) Thermodynamic stability profiles of the same regions as computed by WEB-THERMODYN [12,13], both before (black) and after (red) the deletion. The only effect of this deletion, whose location is indicated by the red arrow, is to displace the downstream profile by 38 bp. However, as shown in (B), the SIDD profile is profoundly altered throughout the region.

Our approach for analyzing duplex destabilization differs fundamentally from others, such as the THERMODYN and MELTMAP algorithms [12–14], which only consider local thermodynamic stability or A+T content. SIDD does not depend on such local properties alone; rather, transitions in superhelical domains are globally interactive. Because strand separation localizes some of the imposed negative superhelicity as untwisting at the open site, it causes a corresponding relaxation that is felt throughout the topological domain. So denaturation at any site will alter the opening probabilities of every other site in the domain. This global coupling can lead to complex interactive transition behaviors that are not reflected by local thermodynamic stability [9]. An example of the long-range coupling induced by superhelicity is shown in Figure 1.

A genome-wide view of destabilization properties offers new perspectives on chromosomal organization and, specifically, on the structural properties of DNA regulatory regions. For yeast these include transcriptional regulatory sites (unpublished data) and the sites regulating the initiation of replication, considered here. SIDD has been implicated in the functioning of replication origins in a variety of organisms. The unique replication origin in *E. coli, oriC,* is superhelically destabilized, and this destabilization has been implicated in its function [15]. Other work has documented pathological origin activity at SIDD sites created by expansion of the pentameric repeat, causing spinocerebellar ataxia type 10 [7]. A role has been established for SIDD in the function of the Epstein–Barr *oriP* origin [16]. Here we focus on developing a genome-wide view of yeast replication origins.

## Results

**Figure 2:**
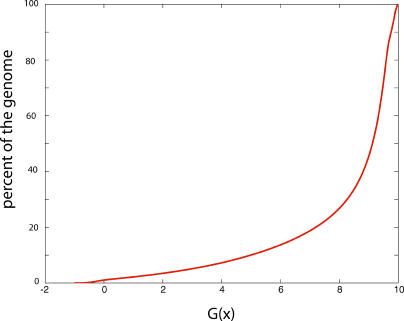
The Cumulative Distribution of Destabilization Levels *G*(*x*) for the Entire Yeast Genome For each value of *G* on the horizontal axis, this curve plots the number of base pairs (expressed as a percent of the genome) needing that amount of free energy (or less) to strand separate. *G* = 10 kcal/mol is sufficient to open any base pair in the genome.

SIDD analysis of the complete yeast genome has been performed under the conditions described in the Materials and Methods section. The cumulative distribution of *G*(*x*) is shown in Figure 2. One sees that most of this genome is not significantly destabilized; half of the base pairs have *G*(*x*) greater than 9.13 kcal/mol. Only 7.23% of the base pairs have *G*(*x*) less than 4 kcal/mol under these conditions, while just 3.48 % have *G*(*x*) less than 2 kcal/mol, indicative of substantial destabilization. Moreover, the significantly destabilized sites are largely confined to regulatory regions governing either transcription (unpublished data) or replication.

### The SIDD Properties of ARS Elements

An exhaustive literature search found 39 experimentally well-characterized ARS elements. These are relatively short regions that function as replication origins in a standard in vivo plasmid assay. The site within each element that acts as the replication origin under these circumstances is usually not more precisely identified.

**Figure 3:**
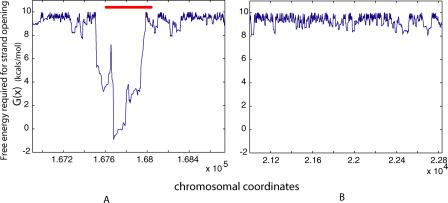
ARS Elements Are More Destabilized Than Other Parts of the Genome (A) The SIDD profile of a region (bp 166936–168740) of Chromosome 6, containing ARS606 (position marked in red). (B) For contrast, the SIDD profile of a randomly chosen but representative genomic location of equal length (Chromosome 6, bp 21000–22834). Graphs of all 39 ARS elements are presented in Protocol S1.

Visual examination of the SIDD profiles of the genomic locations containing these ARS elements shows that the specific ARS sites occur at positions having low *G*(*x*) values, and hence are highly susceptible to destabilization by superhelical stress and thereby unusually prone to strand separation. A representative example is presented in Figure 3. (A complete list of these elements and the SIDD profiles of regions containing each element are presented in Table S1 and Protocol S1, respectively.)

To quantify this propensity, we determined the minimum value *G_min_* of *G*(*x*) occurring within each ARS element. For comparison, at each ARS element we also found *G_min_* in two nearby segments, each the same length as the ARS element, and located symmetrically to either side of it. Here we used comparison regions separated from the ARS element by 250 bp, but equivalent results were found when the comparison regions were chosen to directly abut the ARS elements (data not shown).

**Figure 4:**
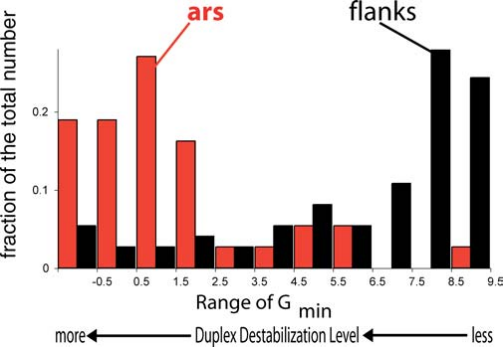
Duplex Destabilization at ARS Elements Compared with Duplex Destabilization at the Surrounding Sequences This histogram shows the distributions of ARS elements (red), and of comparison regions (black) whose *G_min_* values fall in the indicated ranges. (Here, as elsewhere, the lower the *G_min_* value, the more destabilized the region.) The comparison regions were chosen to have the same lengths as the ARS element they flank, and to be positioned 250 bp away from it on either side. There being twice as many comparison regions as ARS elements, these distributions are normalized to show the fraction of sites of each type falling within each interval. Equivalent results were obtained when the comparison regions were chosen to directly abut the ARS elements, so the localization of destabilization at ARS elements is not simply a consequence of their positions within intergenic regions. (ARS elements 302, 303, and 320 on Chromosome III were positioned very close [20 bp separate ARS302 from ARS303, and ARS320 directly abuts ARS303], so for the purpose of these statistical tests these three were regarded as a single site.)

The average value of *G_min_* within these ARS elements was 1.51 kcal/mol, while *G_min_* within the comparison regions averaged 6.29 kcal/mol. It follows that ARS elements are much more susceptible to SIDD than are neighboring regions. The distributions of *G_min_* values within the ARS elements and in their comparison regions are shown in Figure 4.

We next compared the distribution of *G_min_* values within the ARS elements to the genome-wide SIDD distribution shown in Figure 2. Just 2.79% of the base pairs in this genome were destabilized at the level *G*(*x*) less than 1.51 kcal/mol, the average *G_min_* for the ARS elements. This clearly shows that sites that are superhelically destabilized to the extent found at ARS elements are not common.

### The Statistical Significance of this Association

We performed a Wilcoxon–Mann–Whitney rank sum test [17] to rigorously assess the statistical significance of this observed difference in destabilization between ARS elements and their comparison regions. The results show that the null hypothesis (that these distributions are the same) must be rejected with very high confidence—the *p*-value calculated by this test was *p* = 4.28 × 10^−10^. A Kolmogorov–Smirnov test [18] of the same distributions, performed for the same purpose, yielded *p* = 2.91 × 10^−10^. Together, these two nonparametric tests show that the greater destabilization within ARS elements relative to their flanking regions is statistically highly significant.

## Discussion

We have shown that a strong susceptibility to destabilization under stress is a statistically significant attribute of ARS elements, as evaluated in their genomic contexts within the *S. cerevisiae* genome. The fact that 38 of the 39 analyzed ARS elements are significantly destabilized, and on average are much more destabilized than their neighborhoods, makes this one of the most highly conserved attributes known to occur at ARS elements.

Although the SIDD results reported here are consistent with earlier studies of duplex unwinding elements (DUEs) in ARS elements [4], we note three significant differences. First, unlike the attribute of helical stability used to characterize DUEs, SIDD properties are acutely dependent not just on the ARS element sequence itself, but also on its larger context. So altering nearby sequences can drastically change the SIDD properties of a region. This effect is consistent with the observation that origin activity varies depending on chromosomal location, suggesting the influence of local chromatin structure [1]. Second, in addition to finding unwinding regions, SIDD calculations also identify locations where imposed superhelicity diminishes the energy needed to separate the DNA into single strands. This has an exponential effect on the ease with which other molecules can induce strand separation there (see Materials and Methods). Third, unlike DUEs, the destabilization at ARS element locations is not confined to discrete or specific positions within the element.

The presence of stress-destabilized sites at ARS elements has clear implications for the mechanisms of initiation of DNA replication. Under certain circumstances, the presence of a SIDD site alone has been shown to confer a degree of origin activity on an otherwise inactive region [7]. Other more complex roles also are possible. Observations of SIDD near promoters have shown that protein binding can exert regulatory effects by translocating destabilization from the binding site to other locations [11]. Similar events could occur during origin function. The reported dual roles for B2 elements within the ARS element, as being involved either in duplex unwinding [4] or protein binding [19], could be reconciled if protein binding to a destabilized B2 element were to cause a similar regulatory translocation. If the destabilization were to move to the position where unwinding is required for initiation, this could be the mechanism by which binding activates initiation.

To experimentally investigate the details of the role that SIDD may play in the regulation of specific replication origins, the destabilization properties of a region can be altered without changing its base sequence. This involves inserting at another location a DNA sequence that is also susceptible to some type of superhelical transition [9,20]. Since stresses couple together the transition behaviors of all base pairs that experience them, introducing a new competitive region will change the SIDD propensity of the site of interest. This strategy has been used previously to prove that SIDD is involved in the activation of the *ilv*P_G_ promoter of *E. coli* [20].

The complete *S. cerevisiae* genome has been estimated to contain between 200 and 400 ARS elements [1]. The regions of Chromosomes III, VI, and XIV that have been systematically examined for ARS element sites together constitute 6% of the genome and contain 31 ARS elements [21]. If this density is representative, it would give a slightly higher estimate of approximately 500 ARS elements in this genome. Whichever number is used, it is clear that only a small fraction of the ARS elements in yeast have been located to date.

**Figure 5:**
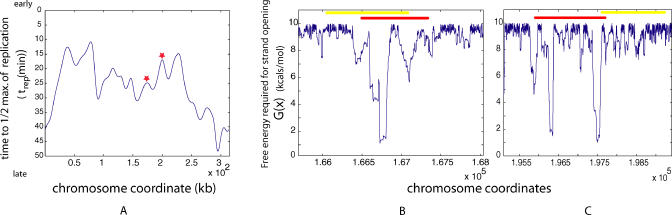
Localization of ARS Elements (A) Replication timing profile of Chromosome 3. The two peaks indicated by red stars are predicted with high confidence to contain replication origins. (Data replotted from [23].) (B and C) SIDD profiles of the two peak regions (from [A]) are plotted to high resolution, along with locations of the known ARS element (red) and the DNA segments within which ORC and MCM proteins were shown to bind [22] (yellow). (B) shows the profile around ARS 310, and (C) shows that of ARS 314.

The statistically highly significant association of SIDD properties with ARS elements reported here suggests that these properties may be useful for finding the precise locations of ARS elements within regions of the yeast genome that are suspected to contain them. Two recent studies identified several such regions on a genome-wide scale. The first study identified DNA segments that showed binding activity for ORC and MCM proteins [22], while the second measured the time of replication across complete chromosomes using density transfer and microarray hybridization [23]. The regions identified by these approaches are roughly 1 kb and 10–20 kb in size, respectively—too large to unambiguously locate ARS elements within them. Since SIDD properties can be calculated with single base pair resolution, predictions of the susceptibility to superhelical destabilization could be used in conjunction with these results to identify potential replication origins throughout the yeast genome. Two illustrative examples are shown in Figure 5. Alternatively, SIDD properties could be used in conjunction with other computational methods (e.g., sequence-based algorithms [24]) of origin prediction to locate potential origins with greater confidence and accuracy.

We have shown that strong susceptibility to destabilization under stress is a highly conserved attribute of ARS elements in *S. cerevisiae.* Our ongoing research suggests that an enhancement of SIDD propensities might also correlate with replication origin locations in higher eukaryotes.

## Materials and Methods

We analyzed the SIDD properties of the complete genome of the S288C strain of *S. cerevisiae* [25]. We used the method described previously whereby the DNA sequence of each chromosome is partitioned into overlapping windows and each window is analyzed separately [5]. Each window (except perhaps the last) has length *N* = 5,000 bp, with successive windows offset by 500 bp so each internal base pair appears in ten windows. The final values of the probability *p*(*x*) and the destabilization energy *G*(*x*) for the base pair at position *x* are calculated as the weighted averages of their computed values in each of the windows that contain that base pair. A detailed description of this algorithm has been presented elsewhere [5].

In these calculations all conformational and free energy parameters are given their experimentally measured values, so there are no free parameters [8,9]. Here we use values appropriate to a temperature of 37 °C and a [Na^+^] of 0.01 M, the conditions of the Kowalski nuclease digestion procedure by which superhelical denaturation is most accurately evaluated [26]. We use superhelix density σ = −0.055, a moderate physiological value [27]. These calculations robustly predict the locations where destabilization occurs, although the details of the transition profiles vary somewhat with assumed conditions. In particular, elevated temperature and increased negative superhelicity act synergistically; higher stress is required to achieve a given level of destabilization at lower temperatures, other factors remaining fixed. 

This analysis of the complete yeast genome required approximately 12 hours to execute on a 28-node Apple X-Serve cluster, each node containing dual 1 GHz G4 processors. The profile of the entire genome is available on request.

To understand the significance of the destabilization energy, consider a system that can assume multiple states, each with an energy *G.* Suppose two specific states, which we call 1 and 2, have energies *G_1_* and* G_2_,* respectively. At equilibrium the ratio of the number of molecules in each state will vary exponentially with the difference in their energies according to *f_1_*/*f_2_* = *exp* [−(*G_1_ −* *G_2_*)/*RT*], where *RT* = 0.616 kcal/mol at a temperature *T* of 37 °C. It follows from this equation that lower energy states are exponentially more highly populated than are higher energy states at equilibrium.

Now, suppose this equilibrium involves the opening of a specific region of DNA by a reversible reaction with another molecule. Let the free energy required for opening this region be *G_1_* in a supercoiled molecule, and *G_2_* in a relaxed molecule, the difference being the destabilization caused by the superhelicity. If this difference is 4.78 kcal/mol (which is the average difference between the ARS elements and their comparison regions), this will favor the open state by *f_1_*/*f_2_* = 2,334, so at equilibrium opening will occur more than 2,000 times as often when this region is superhelically destabilized than when it is not, other factors remaining fixed. We note that this amount of destabilization would bring our *G*(*x*) from 10 kcal/mol just down to 5.2 kcal/mol, which is less than what would be needed to open the region completely. If strand separation at this site is the rate-limiting step in the initiation of a process, this amount of stress-induced destabilization can have a profound effect on the frequency of initiation.

## Supporting Information

Protocol S1 Database of Known ARS Sites and SIDD Profiles of All 39 ARS Elements (2.3 MB PDF).

Table S1 Table of Experimentally Well-Characterized ARS Elements and Associated Free Energies of Destabilization (117 KB DOC).

## Abbreviations

ARS element: autonomously replicating sequence
DUE: duplex unwinding element
SIDD: superhelically induced duplex destabilization

## References

[pcbi-0010007-b01] Newlon CS, DePamphilis ML (1996). DNA replication in yeast. DNA replication in eukaryotic cells.

[pcbi-0010007-b02] Marahrens Y, Stillman B (1992). A yeast chromosomal origin of DNA replication defined by multiple functional elements. Science.

[pcbi-0010007-b03] Huang RY, Kowalski D (1993). A DNA unwinding element and an ARS consensus comprise a replication origin within a yeast chromosome. EMBO J.

[pcbi-0010007-b04] Natale D, Umek R, Kowalski D (1993). Ease of DNA unwinding is a conserved property of yeast replication origins. Nucleic Acids Res.

[pcbi-0010007-b05] Benham CJ, Bi C (2004). The analysis of stress-induced duplex destabilization in long genomic DNA sequences. J Comp Biol.

[pcbi-0010007-b06] Benham CJ (1992). Energetics of the strand separation transition in superhelical DNA. J Mol Biol.

[pcbi-0010007-b07] Potaman VN, Bissler JJ, Hashem VI, Oussatcheva EA, Lu L (2003). Unpaired structures in SCA10 (ATTCT)n.(AGAAT)n repeats. J Mol Biol.

[pcbi-0010007-b08] Benham CJ (1993). Sites of predicted stress-induced DNA duplex destabilization occur preferentially at regulatory loci. Proc Natl Acad Sci U S A.

[pcbi-0010007-b09] Benham CJ (1996). Duplex destabilization in superhelical DNA is predicted to occur at specific transcriptional regulatory regions. J Mol Biol.

[pcbi-0010007-b10] Fye RM, Benham CJ (1999). Exact method for numerically analyzing a model of local denaturation in superhelically stressed DNA. Phys Rev E Stat Phys Plasmas Fluids Relat Interdiscip Topics.

[pcbi-0010007-b11] Sheridan SD, Benham CJ, Hatfield GW (1998). Activation of gene expression by a novel DNA structural transmission mechanism that requires supercoiling-induced DNA duplex destabilization in an upstream activating sequence. J Biol Chem.

[pcbi-0010007-b12] Huang Y, Kowalski D (2003). WEB-THERMODYN: Sequence analysis software for profiling DNA helical stability. Nucleic Acids Res.

[pcbi-0010007-b13] Huang Y, Kowalski D (2003). WEB-THERMODYN [Web-based computer program].

[pcbi-0010007-b14] Lerman LS, Silverstein K (1987). Computational simulation of DNA melting and its application to denaturing gradient gel electrophoresis. Methods Enzymol.

[pcbi-0010007-b15] Kowalski D, Eddy MJ (1989). The DNA unwinding element: A novel, *cis*-acting component that facilitates opening of the *Escherichia coli* replication origin. EMBO J.

[pcbi-0010007-b16] Polonskaya Z, Benham CJ, Hearing J (2004). Role for a region of helically unstable DNA within the Epstein-Barr virus latent cycle origin of DNA replication in origin function. Virology.

[pcbi-0010007-b17] DeGroot MH (1975). Probability and statistics.

[pcbi-0010007-b18] Chakravarti IM, Laha RG, Roy J (1967). Handbook of methods of applied statistics, Volume 1.

[pcbi-0010007-b19] Wilmes GM, Bell SP (2002). The B2 element of the *Saccharomyces cerevisiae* ARS1 origin of replication requires specific sequences to facilitate pre-RC formation. Proc Natl Acad Sci U S A.

[pcbi-0010007-b20] Sheridan S, Benham CJ, Hatfield GW (1999). Inhibition of supercoiling-dependent transcriptional activation by a distant B-DNA to Z-DNA transition. J Biol Chem.

[pcbi-0010007-b21] Newlon CS, Theis JF (2002). DNA replication joins the revolution: Whole-genome views of DNA replication in budding yeast. Bioessays.

[pcbi-0010007-b22] Wyrick JJ, Aparicio JG, Chen T, Barnett JD, Jennings EG (2001). Genome-wide distribution of ORC and MCM proteins in *S. cerevisiae:* High-resolution mapping of replication origins. Science.

[pcbi-0010007-b23] Raghuraman MK, Winzeler EA, Collingwood D, Hunt S, Wodicka L (2001). Replication dynamics of the yeast genome. Science.

[pcbi-0010007-b24] Breier AM, Chatterji S, Cozzarelli NR (2004). Prediction of *Saccharomyces cerevisiae* replication origins. Genome Biol.

[pcbi-0010007-b25] Stanford University School of Medicine Department of Genetics (2004). *Saccharomyces* Genome Database [database].

[pcbi-0010007-b26] Kowalski D, Natale DA, Eddy MJ (1988). Stable DNA unwinding, not “breathing,” accounts for single-strand-specific nuclease hypersensitivity of specific A+T-rich sequences. Proc Natl Acad Sci U S A.

[pcbi-0010007-b27] Kouzine F, Liu J, Sanford S, Chung HJ, Levens D (2004). The dynamic response of upstream DNA to transcription-generated torsional stress. Nat Struct Mol Biol.

[pcbi-0010007-b28] Zaret KS, Sherman F (1982). DNA sequence required for efficient transcription termination in yeast. Cell.

