# Supplementary material for: Susceptibility to Superhelically Driven DNA Duplex Destabilization: A Highly Conserved Property of Yeast Replication Origins
Source: PLoS Comput Biol. 2005 Jun 24;1(1):e7. doi: 10.1371/journal.pcbi.0010007 (PMC1183513; doi:10.1371/journal.pcbi.0010007)
Supplement: Protocol S1 — (2.3 MB PDF). [file pcbi.0010007.sd001.pdf]

Supplementary Material

Database of Known ARS Sites: An exhaustive search of published literature found 39 well characterized and well localized ARS elements in the *S. cerevisiae* genome. In most cases the chromosomal positions of the ARSs were verified by a BLAST search of the current release of the genomic sequence. To date, only chromosomes III and VI, and part of chromosome XIV, have been systematically examined for ARS locations and replication origin activity [reviewed in (*4*)] Most of the ARSs found in our search were from these regions: 19 from chromosome III, 9 from chromosome VI and 3 from chromosome XIV. 8 ARS elements from other chromosomes were also identified(*5*). A table of these 39 elements and their SIDD profiles is included. Comparison regions for G_{min} values were located 250 bp away from the ends of the ARS sites to compensate for the differences in experimental techniques in characterization and localization of ARS. However, we also compared G_{min} values in regions immediately abutting the ARS positions to ensure that our results were not simply a feature of the ARS locations in the intergenic regions

REFERENCES:

1. C. J. Benham, C. Bi, *J. Comp. Biol* **to appear** (2004).
2. http://www.yeastgenome.org/.
3. D. Kowalski, D. A. Natale, M. J. Eddy, *Proc Natl Acad Sci U S A* **85**, 9464-8 (Dec, 1988).
4. C. S. Newlon, J. F. Theis, *Bioessays* **24**, 300-4 (Apr, 2002).
5. ARSs 302, 303 and 320 on chromosome III were positioned very close (20 bp separate ARS302 from ARS303; and ARS320 directly abuts ARS303), so for the purpose of these statistical tests these three were regarded as a single site.

The SIDD profiles of all 39 ARS elements are shown below. G(x), in kcal/mol, is the Free energy needed to ensure that the basepair at position x is open. The genomic locations of the ARS are marked in red.

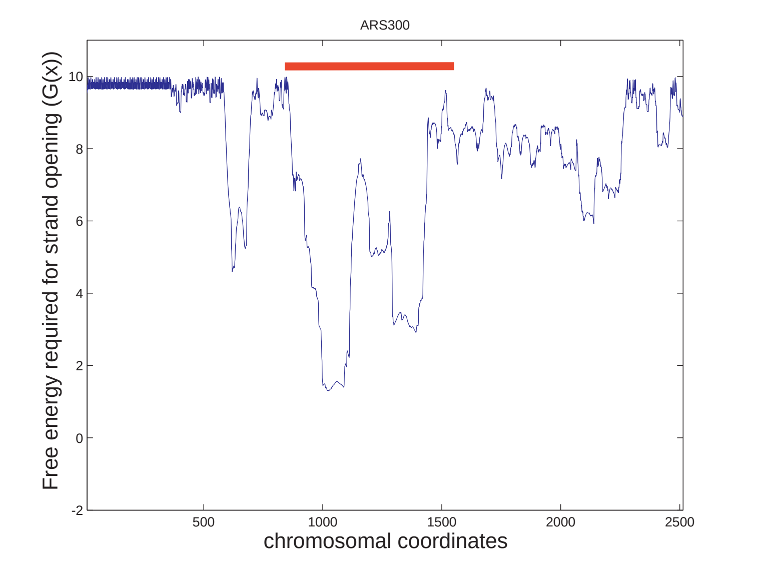
ARS300
Free energy required for strand opening (G(x))
chromosomal coordinates

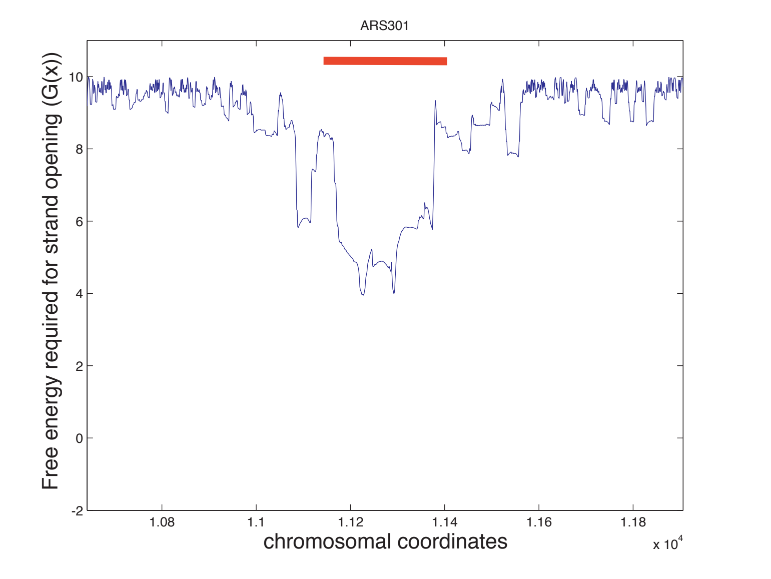
ARS301
Free energy required for strand opening (G(x))
chromosomal coordinates
x 10^4

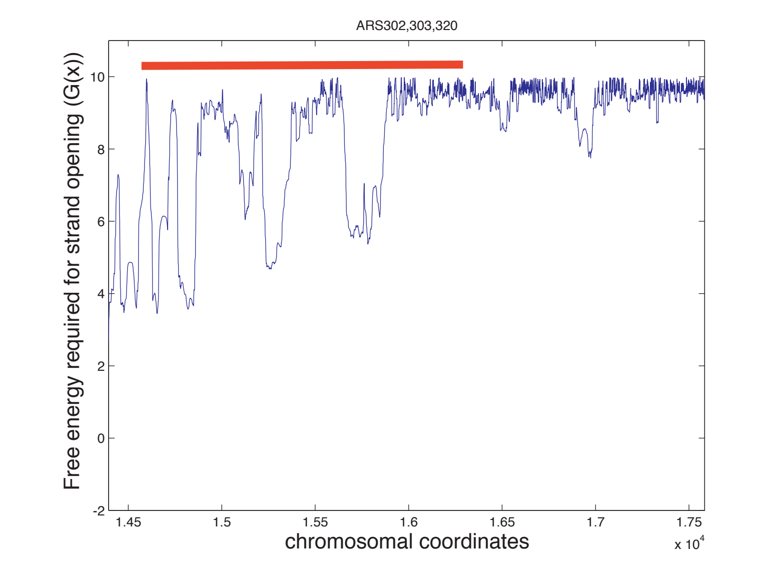
ARS302,303,320
Free energy required for strand opening (G(x))
chromosomal coordinates
x 10^4

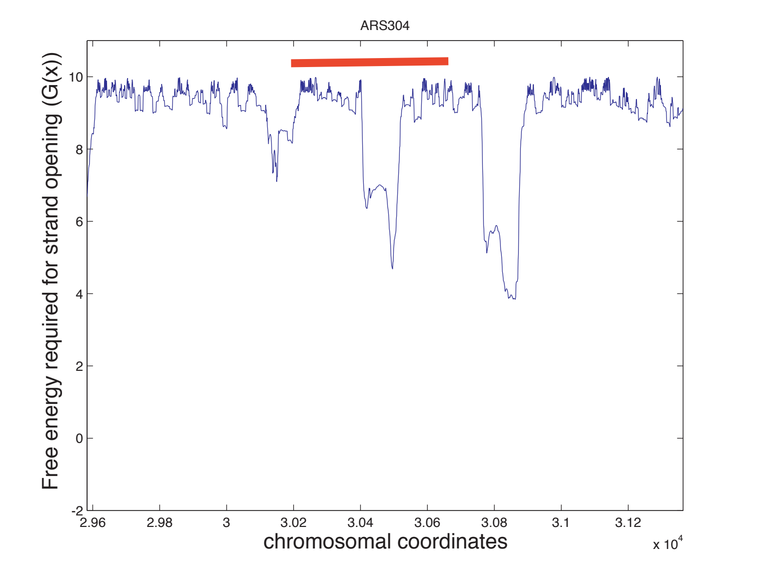
ARS304
Free energy required for strand opening (G(x))
chromosomal coordinates
x 10^4

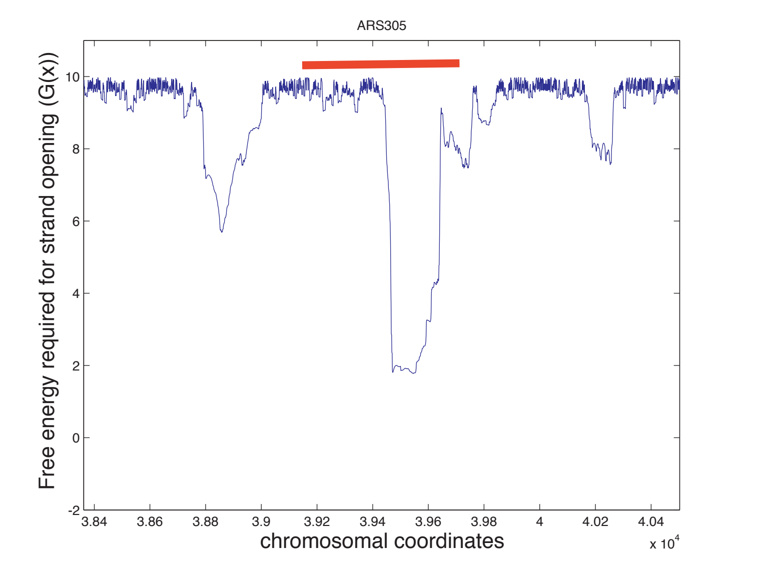
ARS305
Free energy required for strand opening (G(x))
chromosomal coordinates
x 10^4

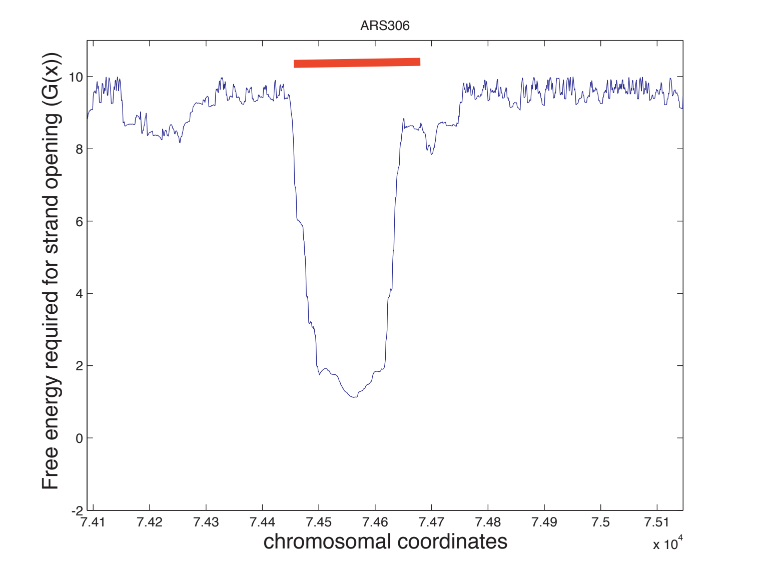
ARS306
Free energy required for strand opening (G(x))
chromosomal coordinates
x 10^4

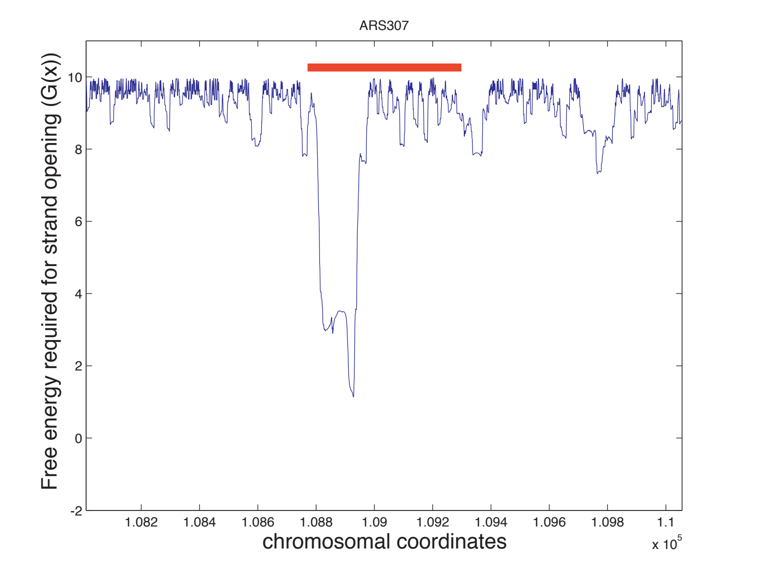
ARS307
Free energy required for strand opening (G(x))
chromosomal coordinates
x 10^5

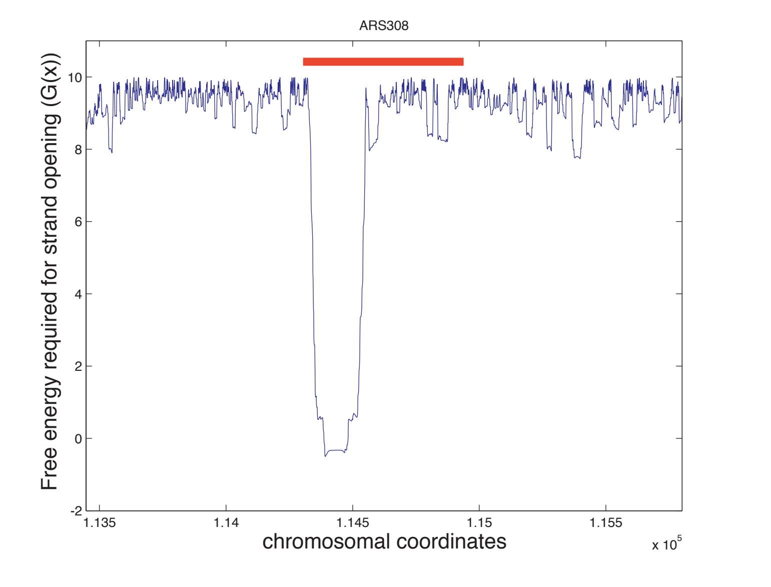
ARS308
Free energy required for strand opening (G(x))
chromosomal coordinates
x 10^5

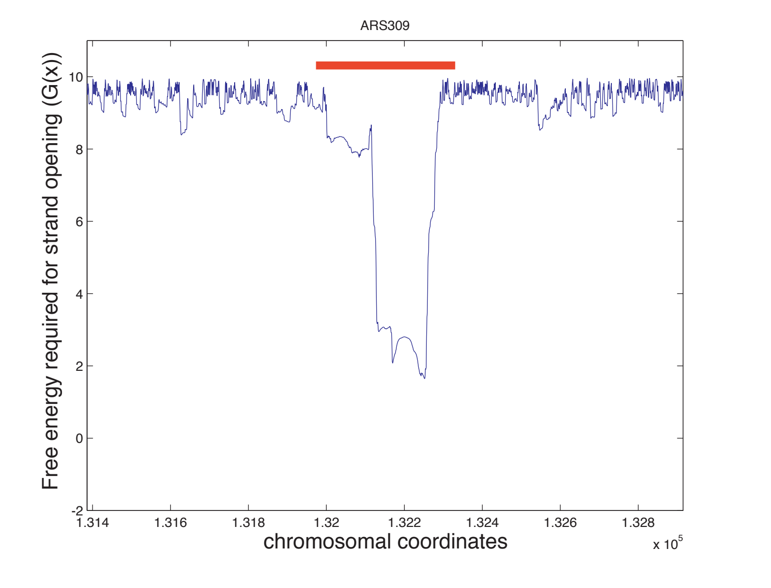
ARS309
Free energy required for strand opening (G(x))
chromosomal coordinates
x 10^5

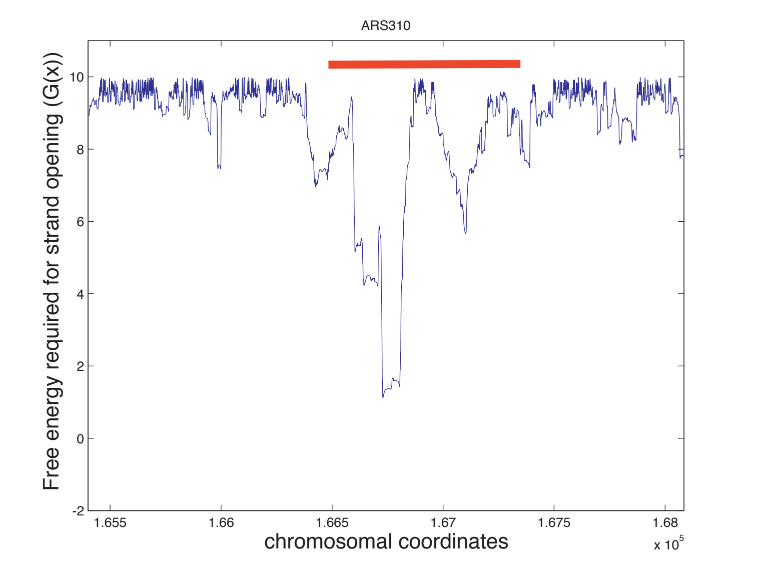
ARS310
Free energy required for strand opening (G(x))
chromosomal coordinates
x 10^5

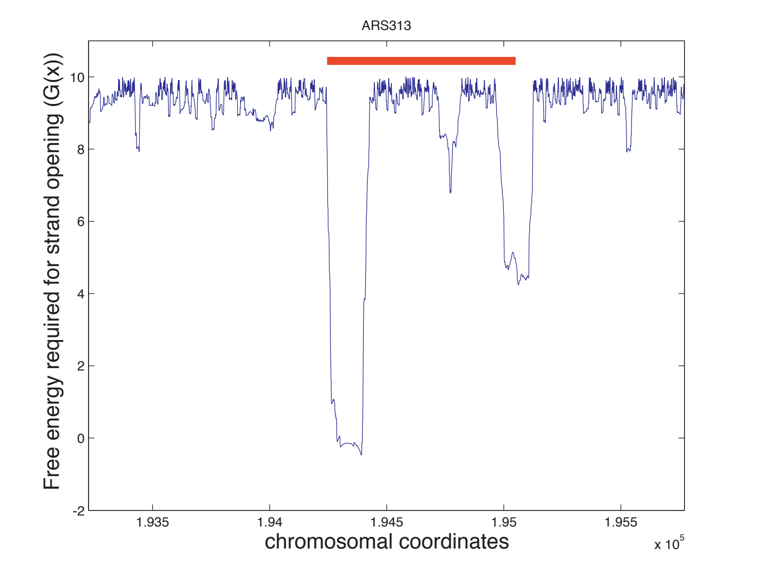
ARS313
Free energy required for strand opening (G(x))
chromosomal coordinates
x 10^5

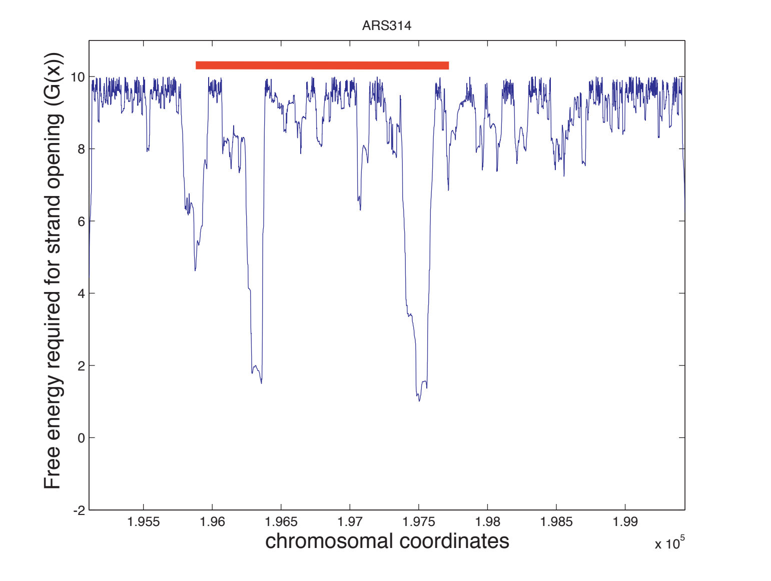
ARS314
Free energy required for strand opening (G(x))
chromosomal coordinates
x 10^5

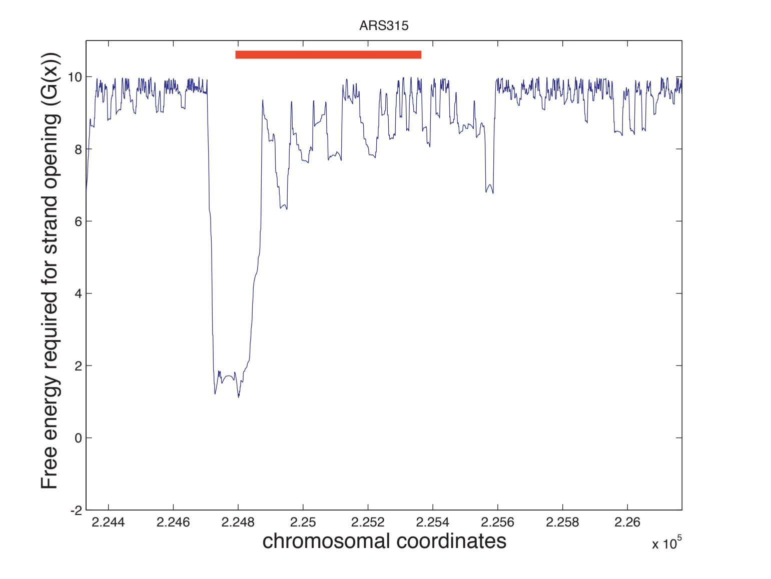
ARS315
Free energy required for strand opening (G(x))
chromosomal coordinates
x 10^5

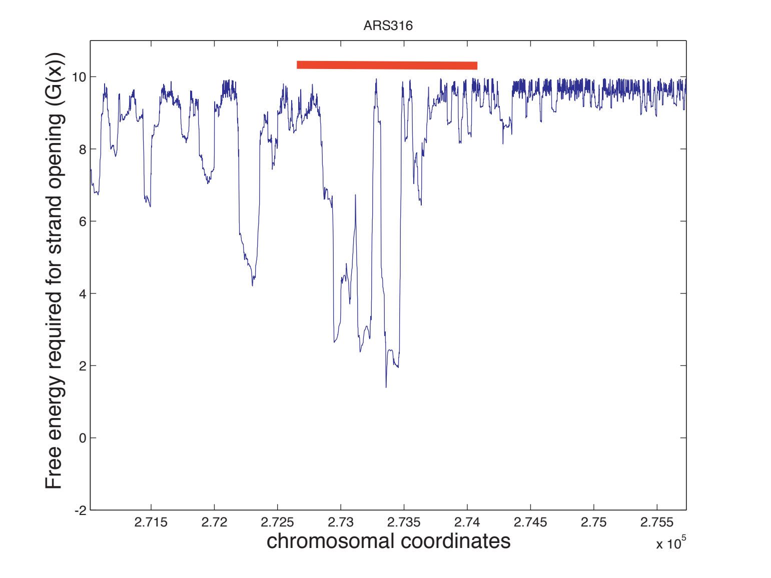
ARS316
Free energy required for strand opening (G(x))
chromosomal coordinates
x 10^5

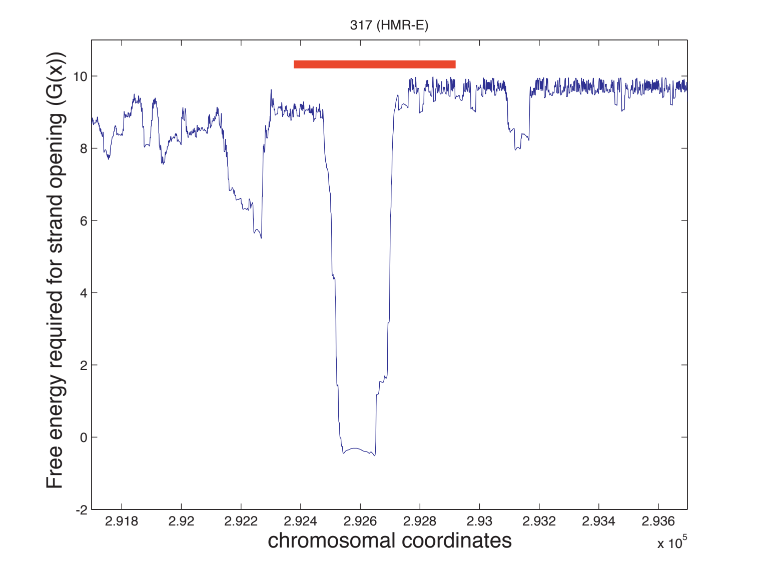
317 (HMR-E)
Free energy required for strand opening (G(x))
chromosomal coordinates
x 10^5

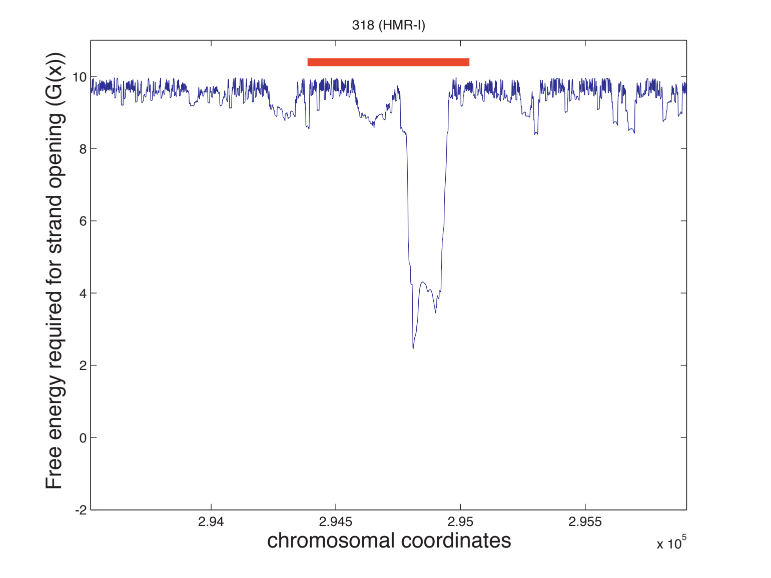
318 (HMR-I)
Free energy required for strand opening (G(x))
chromosomal coordinates
x 10^5

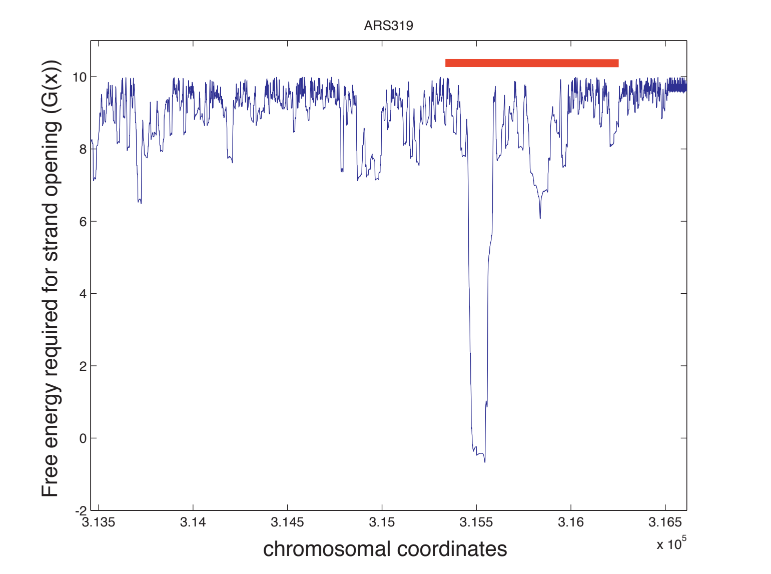
ARS319
Free energy required for strand opening (G(x))
chromosomal coordinates
x 10^5

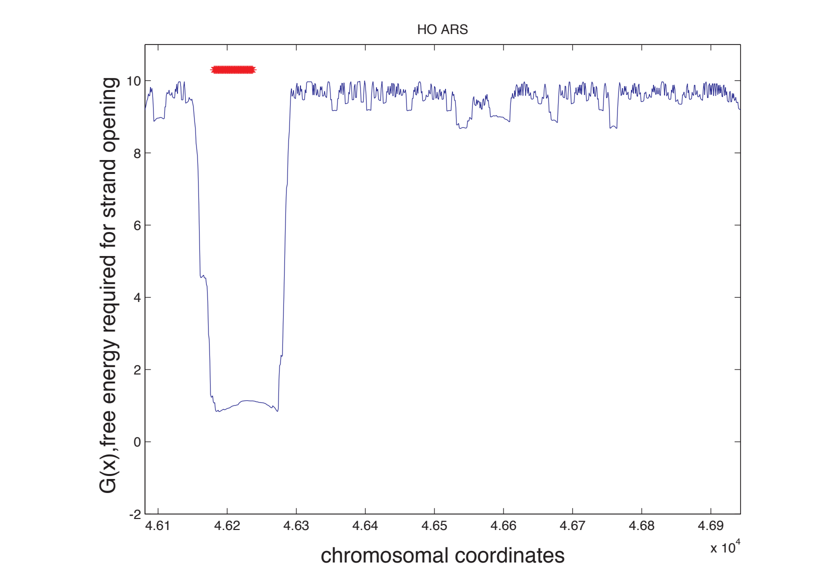
HO ARS
G(x),free energy required for strand opening
chromosomal coordinates
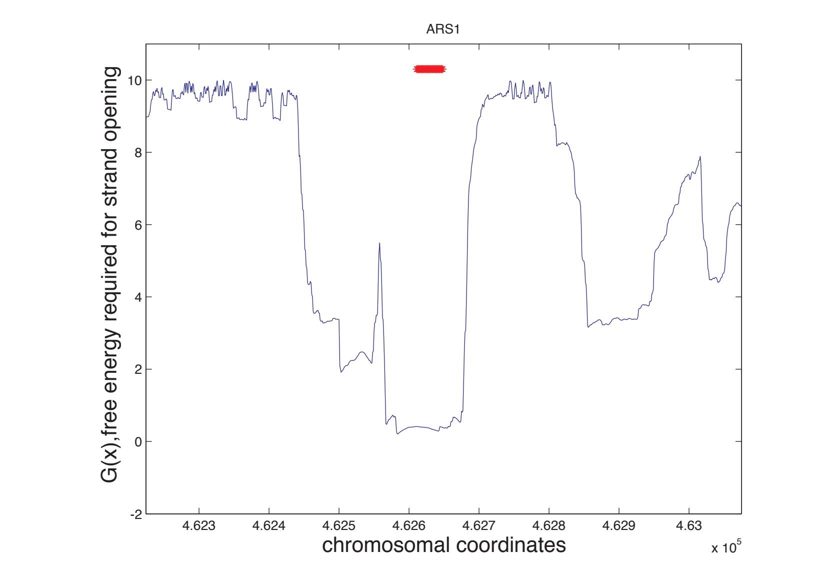
ARS1
G(x),free energy required for strand opening
chromosomal coordinates
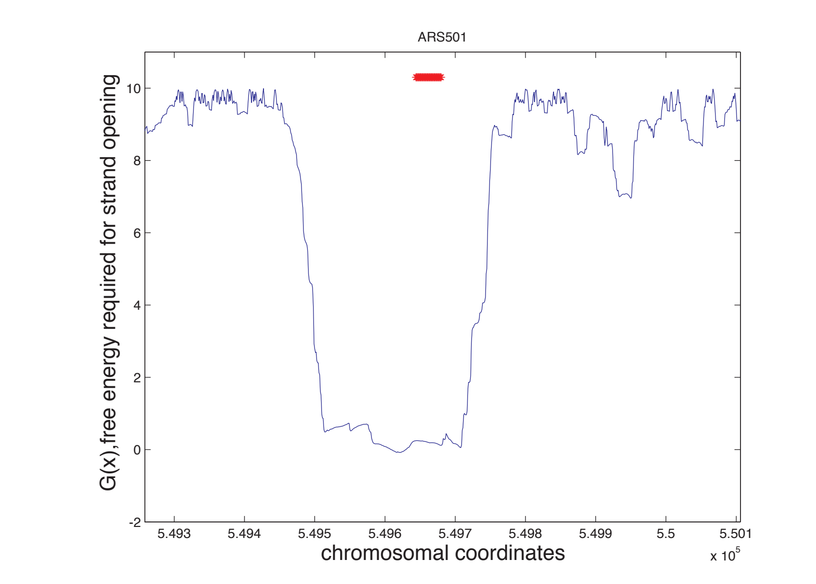
ARS501
G(x),free energy required for strand opening
chromosomal coordinates
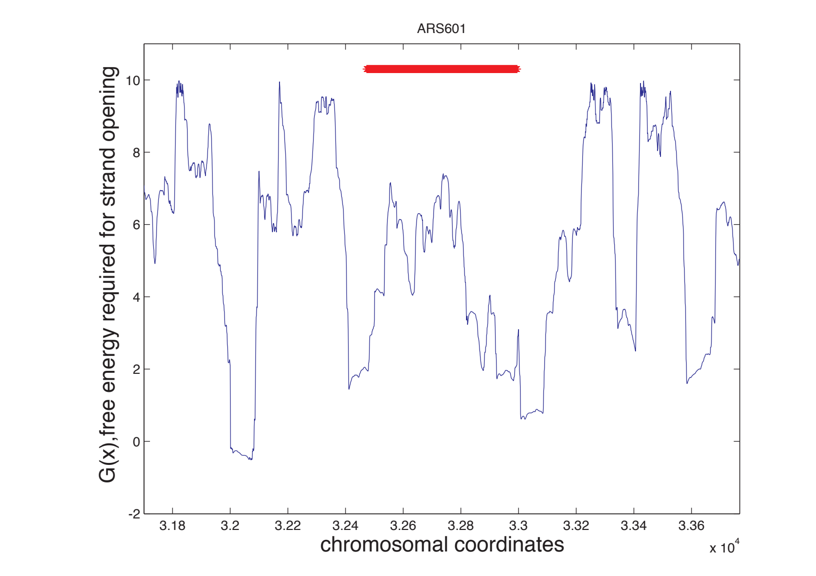
ARS601
G(x),free energy required for strand opening
chromosomal coordinates
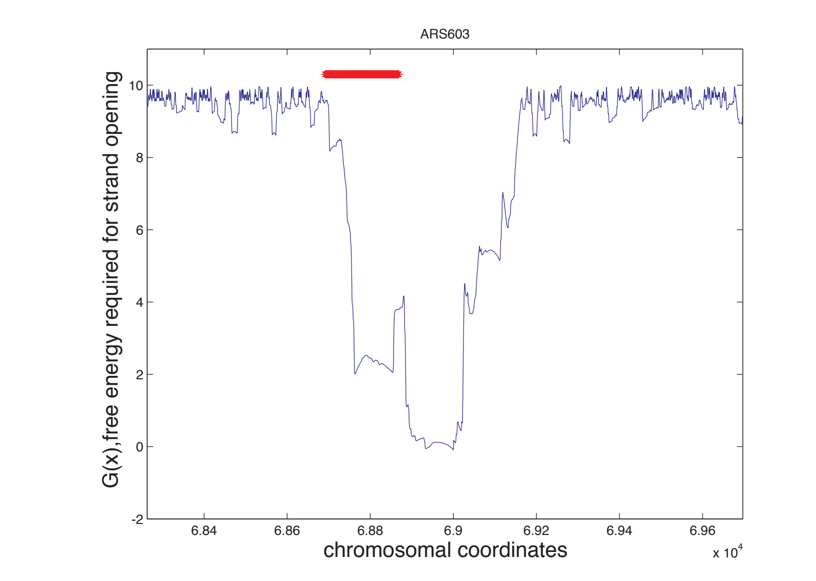
ARS603
G(x),free energy required for strand opening
chromosomal coordinates
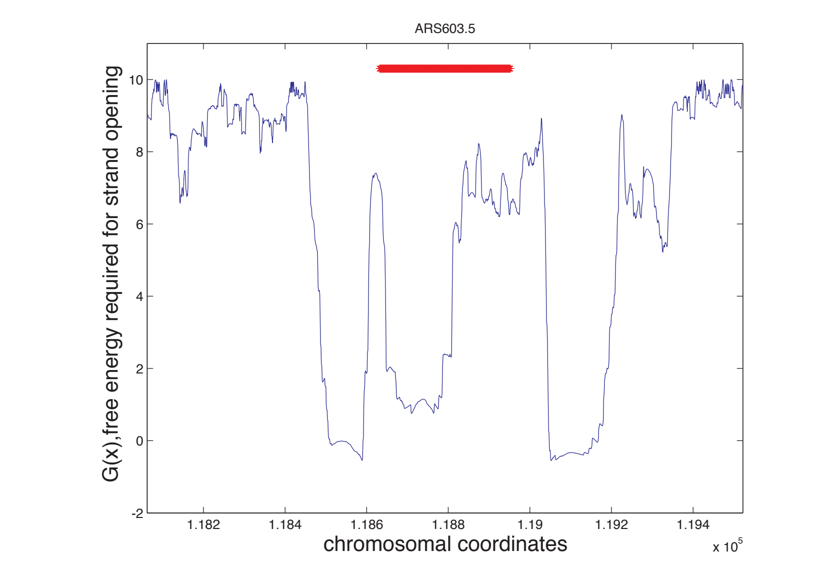
ARS603.5
G(x),free energy required for strand opening
chromosomal coordinates
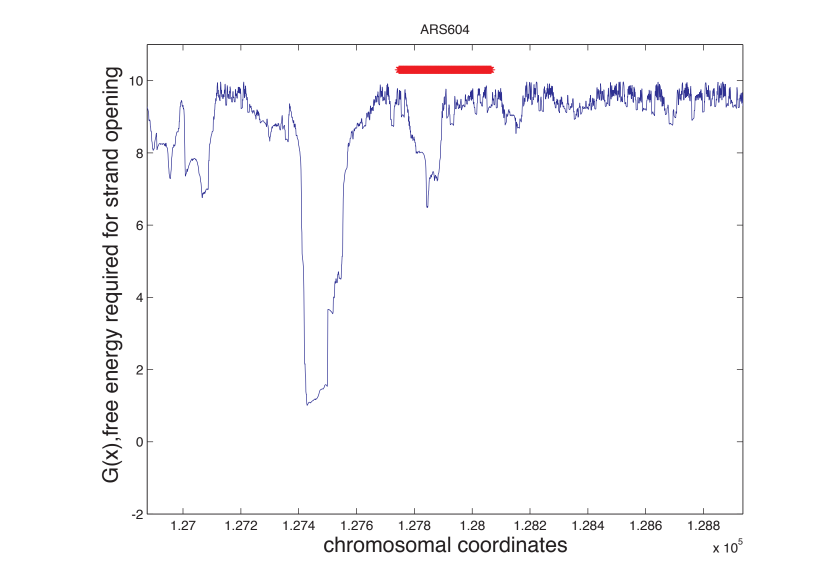
ARS604
G(x),free energy required for strand opening
chromosomal coordinates
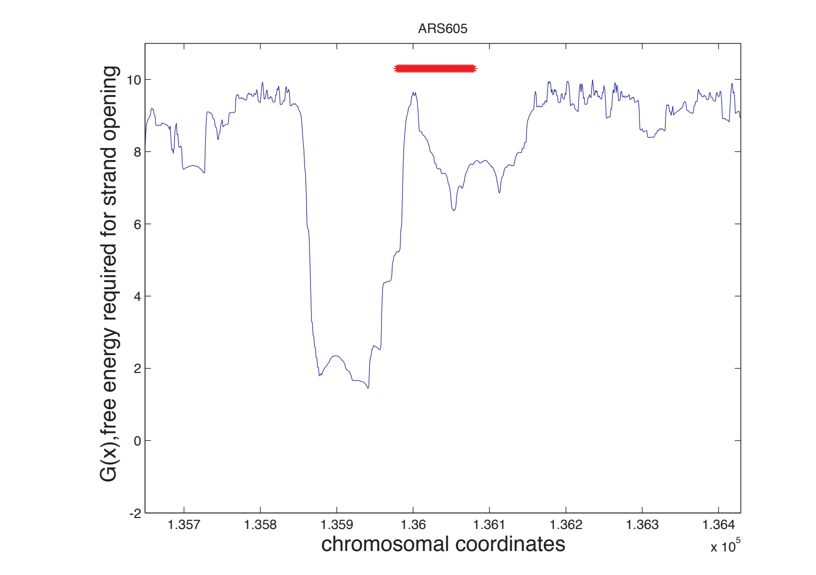
ARS605
G(x),free energy required for strand opening
chromosomal coordinates
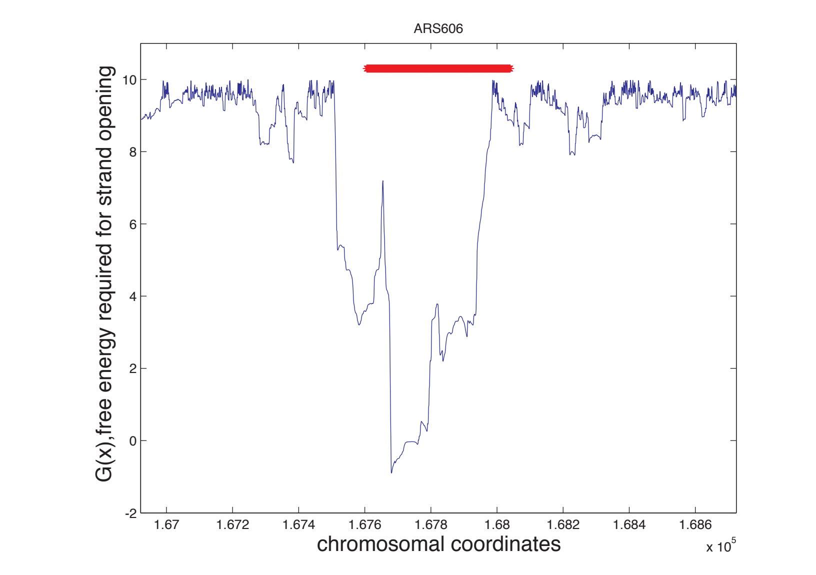
ARS606
G(x),free energy required for strand opening
chromosomal coordinates
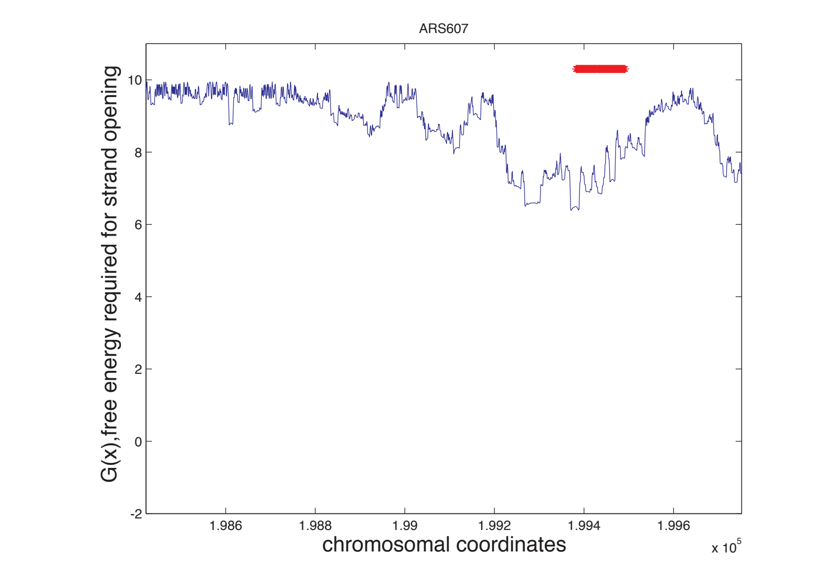
ARS607
G(x),free energy required for strand opening
chromosomal coordinates
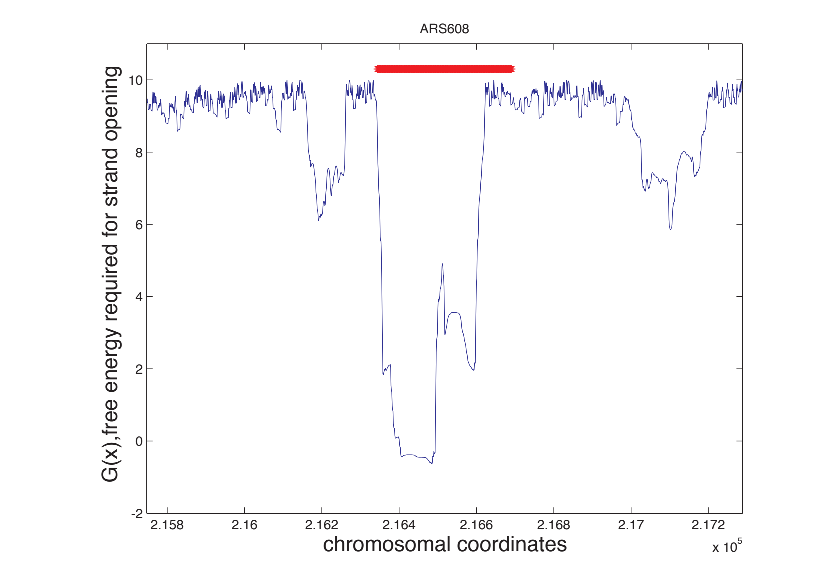
ARS608
G(x),free energy required for strand opening
chromosomal coordinates
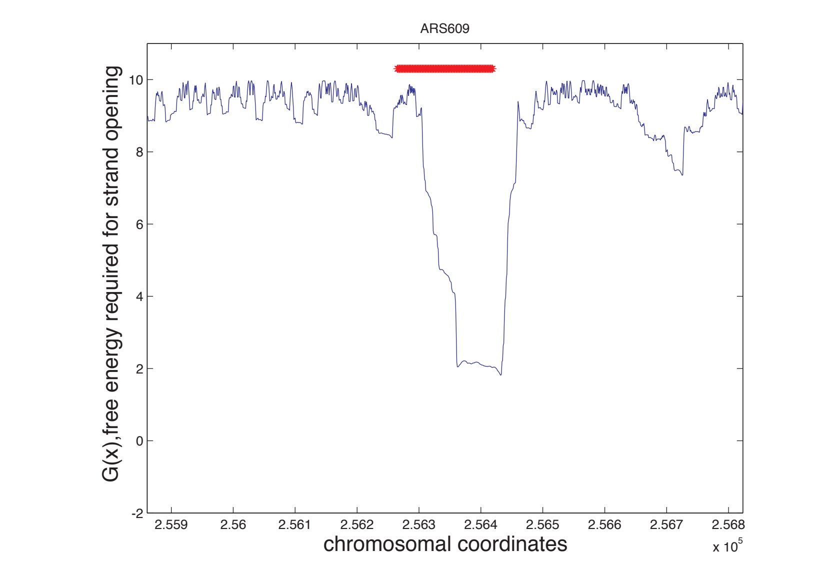
ARS609
G(x),free energy required for strand opening
chromosomal coordinates
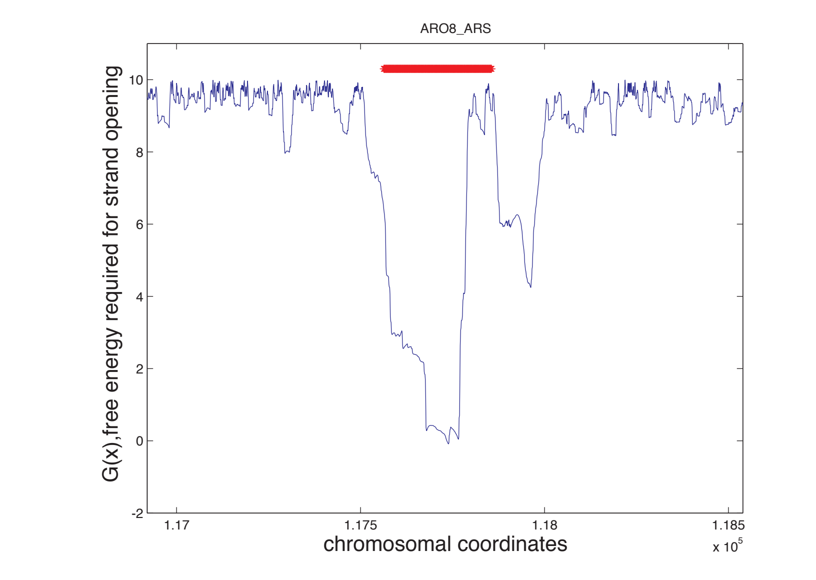
ARO8_ARS
G(x),free energy required for strand opening
chromosomal coordinates
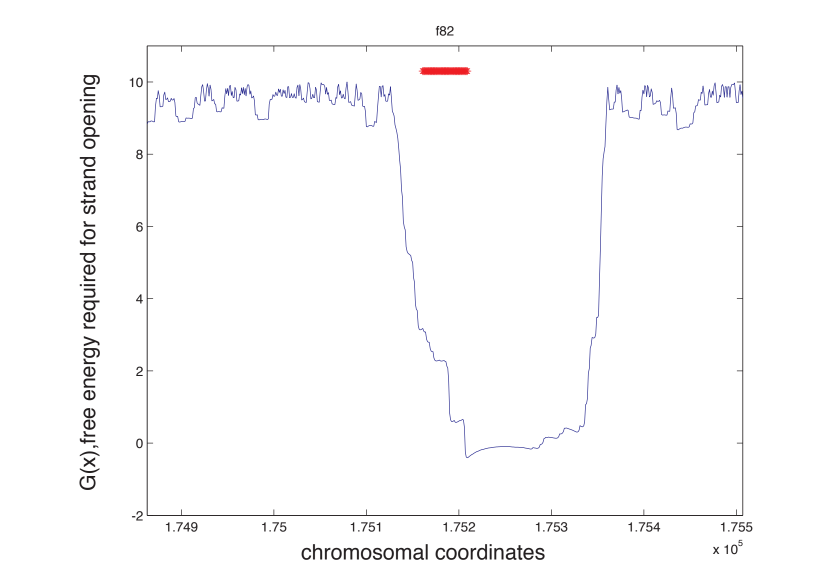
f82
G(x),free energy required for strand opening
chromosomal coordinates
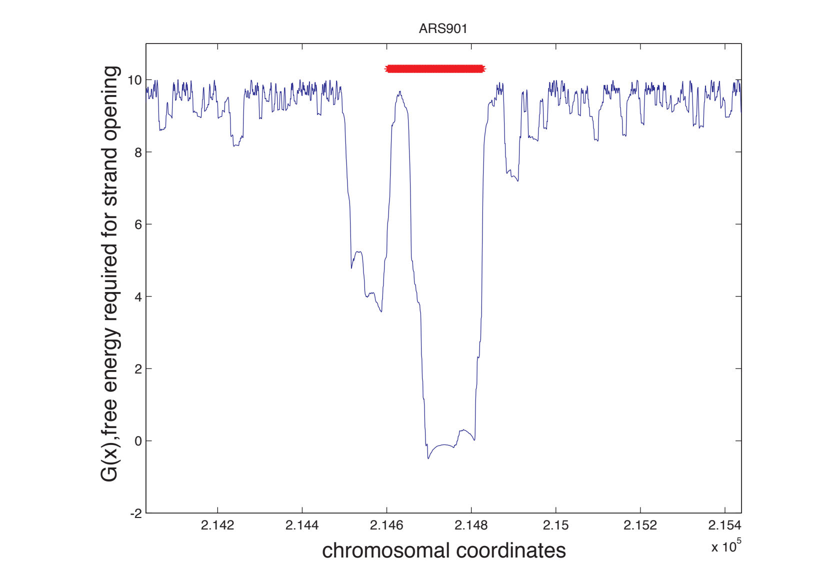
ARS901
G(x),free energy required for strand opening
chromosomal coordinates
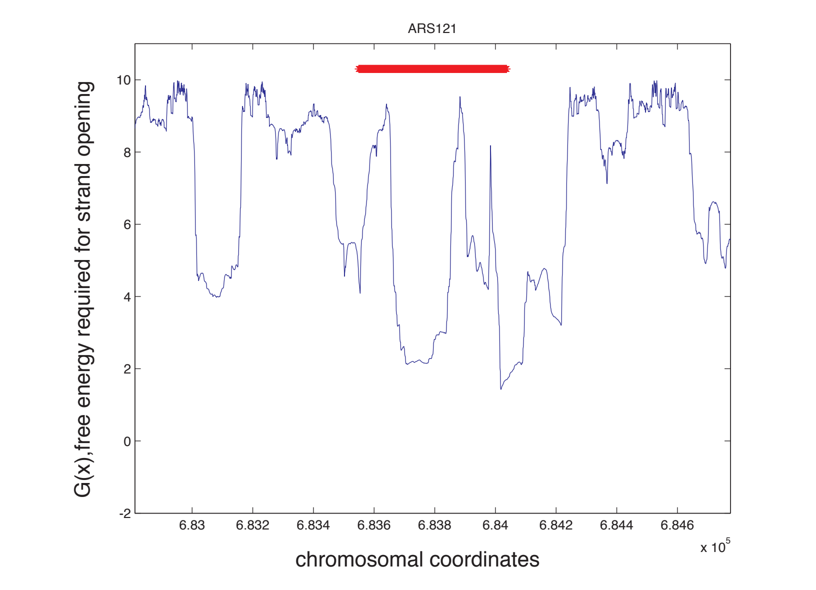
ARS121
G(x),free energy required for strand opening
chromosomal coordinates
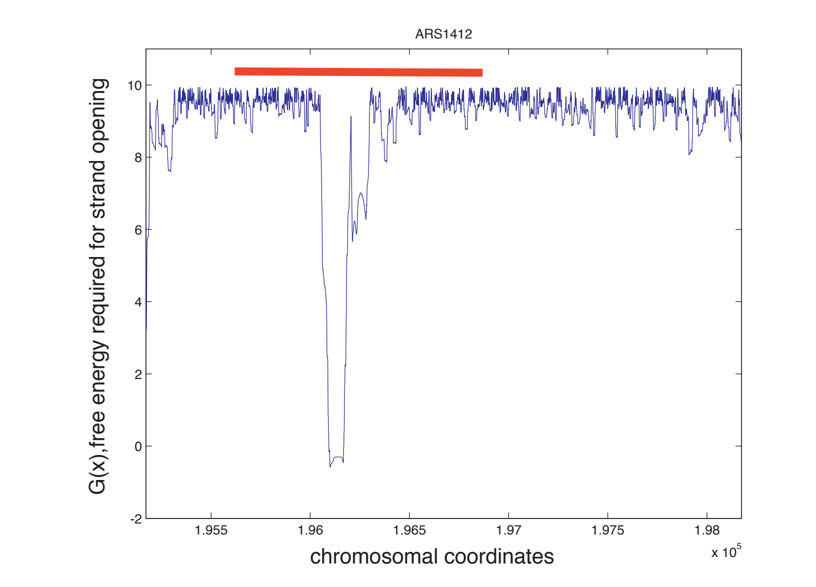
ARS1412
G(x),free energy required for strand opening
chromosomal coordinates
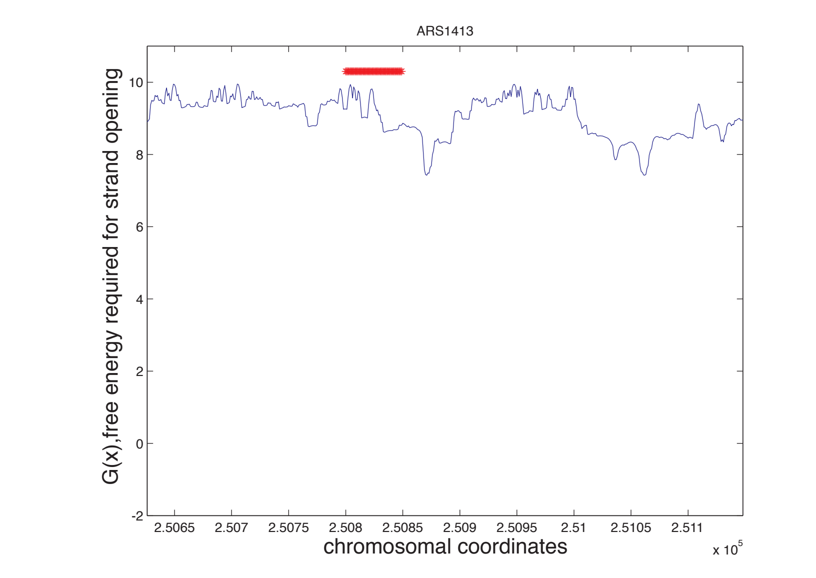
ARS1413
G(x),free energy required for strand opening
chromosomal coordinates
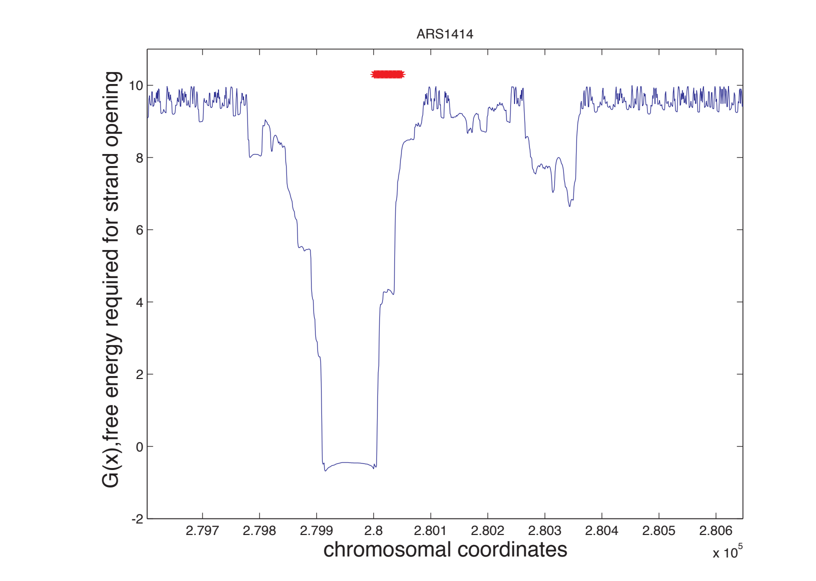
ARS1414
G(x),free energy required for strand opening
chromosomal coordinates
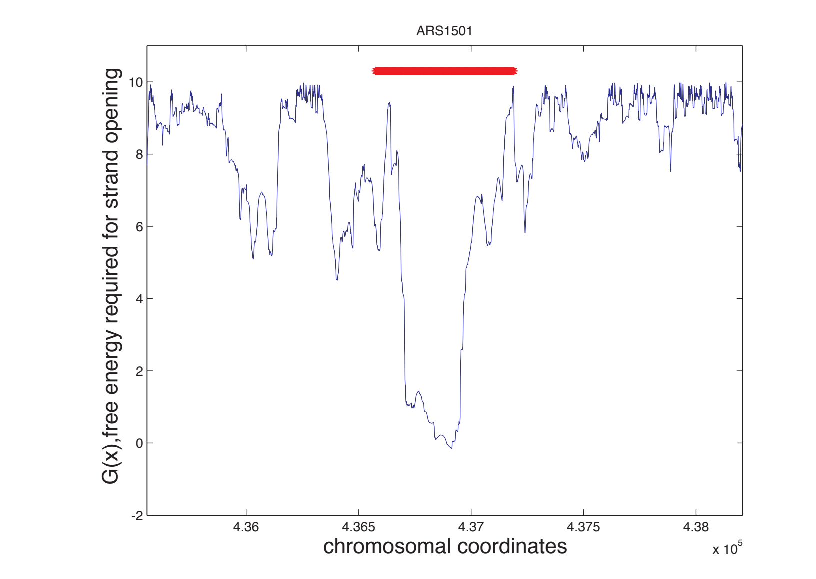
ARS1501
G(x),free energy required for strand opening
chromosomal coordinates
