# Supplementary material for: Susceptibility to Superhelically Driven DNA Duplex Destabilization: A Highly Conserved Property of Yeast Replication Origins
Source: PLoS Comput Biol. 2005 Jun 24;1(1):e7. doi: 10.1371/journal.pcbi.0010007 (PMC1183513; doi:10.1371/journal.pcbi.0010007)
Supplement: Table S1 — (117 KB DOC). [file pcbi.0010007.st001.doc]

Table S1.

| **ARS** | **Chromosome** | **Start Position** | **Stop Position** | **Size** | **Minimum G(x)** | | | **References** |
| --- | --- | --- | --- | --- | --- | --- | --- | --- |
|  |  |  |  |  | **5' End** | **In ARS segment** | **3' End** |  |
| ARS300 | 3 | 838 | 1551 | 714 | 7.768 | 1.3007 | 5.9235 | 1, 4, 5 |
| ARS301 | 3 | 11146 | 11401 | 256 | 8.981 | 3.9438 | 8.6453 | 2, 3, 4, 5 |
| ARS302 | 3 | 14575 | 14849 | 275 | -0.412 | 3.447 | 7.7456 | 2, 3, 4, 5 |
| ARS303 | 3 | 14871 | 15213 | 343 | For the purpose of analysis, ARS 303,320 and 304 |  |  | 2,3,4,5,6 |
| ARS320 | 3 | 15214 | 16274 | 1061 | were treated as one continguous stretch. |  |  | 4, 5, 6 |
| ARS304 | 3 | 30200 | 30657 | 458 | -0.651 | 4.6815 | 8.6178 | 2,3,4,5,7 |
| ARS305 | 3 | 39159 | 39706 | 548 | 5.686 | 1.7774 | 7.5567 | 1, 2, 7, 8, 9 |
| ARS306 | 3 | 74458 | 74677 | 220 | 3.841 | 1.1214 | 9.1061 | 1,2,6,10,11 |
| ARS307 | 3 | 108776 | 109291 | 516 | 8.493 | 1.1355 | 7.3187 | 1,2,7, 10,12,13,14,15 |
| ARS308 | 3 | 114315 | 114933 | 619 | 7.895 | -0.5005 | 7.7392 | 1, 2, 6, 13 |
| ARS309 | 3 | 131979 | 132322 | 344 | 8.387 | 1.6476 | 8.8517 | 1,2,7,13,15 |
| ARS310 | 3 | 166495 | 167340 | 846 | 7.45 | 1.1139 | 4.9236 | 1,2,6,7,20 |
| ARS313 | 3 | 194258 | 195040 | 783 | 7.928 | -0.4695 | 4.6186 | 1, 2, 21 |
| ARS314 | 3 | 195904 | 197704 | 1801 | -0.47 | 1.0081 | -0.588 | 21 |
| ARS315 | 3 | 224800 | 225359 | 560 | 1.107 | 1.1139 | 8.3643 | 21 |
| ARS316 | 3 | 272673 | 274077 | 1405 | 4.204 | 1.391 | 8.3995 | 21 |
| 317 (HMR-E) | 3 | 292381 | 292914 | 534 | 5.498 | -0.5153 | 8.2142 | 21 |
| 318 (HMR-I) | 3 | 294397 | 295027 | 631 | 9.114 | 2.4589 | 8.3829 | 19, 21 |
| ARS319 | 3 | 315350 | 316235 | 886 | 7.117 | -0.675 | 9.0473 | 21 |
| HO ARS | 4 | 46181 | 46237 | 57 | -0.673 | 0.8364 | 8.6728 | 20 |
| ARS1 | 4 | 462611 | 462648 | 38 | 8.905 | 0.2909 | 3.3542 | 21,22 |
| ARS501 | 5 | 549644 | 549680 | 37 | 9.272 | 0.1192 | 6.952 | 23 |
| ARS601 | 6 | 32473 | 32995 | 523 | -0.512 | 1.6766 | 1.5976 | 24,25,26 |
| ARS603 | 6 | 68691 | 68869 | 179 | 8.986 | 2.0084 | 6.0528 | 24,25,26 |
| ARS603.5 | 6 | 118632 | 118952 | 321 | 6.572 | 0.7516 | 3.6788 | 24,25,26 |
| ARS604 | 6 | 127746 | 128066 | 321 | 1.011 | 6.4858 | 8.9281 | 24,25,26 |
| ARS605 | 6 | 135979 | 136079 | 101 | 1.625 | 5.2224 | 8.5852 | 24,25,26,27 |
| ARS606 | 6 | 167606 | 168040 | 435 | 8.183 | -0.8998 | 8.3355 | 24,25,26 |
| ARS607 | 6 | 199382 | 199492 | 111 | 7.947 | 6.3992 | 6.5104 | 24,25,26,27 |
| ARS608 | 6 | 216344 | 216691 | 348 | 8.551 | -0.6302 | 5.8517 | 24,25,26 |
| ARS609 | 6 | 256265 | 256419 | 155 | 8.827 | 2.0298 | 7.3514 | 24,25,26,27 |
| ARSO8 ARS | 7 | 117563 | 117856 | 294 | 7.958 | -0.0857 | 8.4448 | 28 |
| f82 | 9 | 175161 | 175209 | 49 | 8.835 | -0.4059 | 9.363 | 29 |
| ARS901 | 9 | 214603 | 214826 | 224 | 8.155 | -0.4978 | 8.2933 |  |
| ARS121 | 10 | 683328 | 683816 | 489 | 3.976 | 1.4265 | 4.7808 | 30 |
| ARS1412 | 14 | 195652 | 196883 | 1232 | 1.77 | -0.583 | 2.9403 | 31 |
| ARS1413 | 14 | 250800 | 250849 | 50 | 4.533 | 8.6134 | 8.3441 | 31 |
| ARS1414 | 14 | 280001 | 280049 | 49 | 9.352 | -0.5743 | 6.6419 | 31 |
| ARS1501 | 15 | 436575 | 437191 | 617 | 5.089 | -0.15 | 7.5161 | 32 |
| average min G(x) |  |  |  |  | 5.7 | 1.51389 | 6.8936 |  |
|  |  |  |  |  | average of both flanks: |  | 6.2969 |  |

**References**

1 Button, L.L., and C.R. Astell (1988) DNA fragments isolated from the left end of chromosome III in yeast are repaired to generate functional telomeres. Genome **30:**758-765.

2 Newlon, C.S., Lipchitz, L.R., Collins, I., Deshpande, A., Devenish, R.J., Green, R.P., Klein, H.L., Palzkill, T.G., Ren, R., Synn, S., Woody, S.T. (1991). Analysis of a circular derivative of *Saccharomyces cerevisiae* chromosome *III*: A physical map and idÀ
Ø ä
ç Ġ
ģ ĸļ 0ûûNewlon, C.S.*, C*ollins, I., Dershowit**z, A**

3 Newlon, C.S., Collins, I., Dershowitz, A., Deshpande, A.M., Greenfeder, S.A., Ong, L.Y., and Theis, J.F. (1993). Analysis of replication origin function on chromosome III of *Saccharomyces cerevisiae*. Cold Spring Harbor Symp. Quant. Biol. **58**:415-423.

4 Dubey, D.D., L.R. Davis, S.A. Greenfeder, L.Y. Ong, J. Zhu, J.R. Broach, C.S. Newlon, and J.A. Huberman. 1991. Evidence suggesting that the *ARS* elements associated with silencers of the yeast mating type locus, *HML*, do not function as chromosomal DNA rep
 Ô
× đ
ġ Ģĥ 0*ÿÿHuberman, J.A*.**, D**

5 Huberman, J.A., D.D. Dubey, K.A. Nawotka, G. Russev, J.A. Sanchez, Y. Yoon, and M.H.K. Linskens. 1992. Directions of DNA replication in yeast and mammalian cells. In *DNA replication: the regulatory mechanisms* (eds., P. Hughes, et al.), pp. 83. Springer

6 Vujcic, M., C.A. Miller, and D. Kowalski (1999) Activation of silent replication origins at autonomously replicating sequence elements near the *HML* locus in budding yeast. Mol. Cell. Biol. **19:**6098-6109.

7 Theis, J.F., Yang, C., Schaefer, C.B., and Newlon, C.S. (1999) DNA sequence and functional analysis of homologous ARS elements of *Saccharomyces cerevisiae* and *S. carlsbergensis*. Genetics **152:**943-952.

8 Palzkill, T.G., Oliver, S.G., Newlon, C.S. (1986). DNA sequence analysis of *ARS* elements from chromosome III of *Saccharomyces cerevisiae*: Identification of a new conserved sequence. Nucleic Acids Res. **14**:6247-6264.

9 Huberman, J.A., Zhu, J., Davis, L.R., Newlon, C.S. (1988). Close association of a DNA replication origin and an *ARS* element on chromosome III of the yeast, *Saccharomyces cerevisiae*. Nucleic Acids Res. **16**:6373-6384.

10 Huang, R.Y. and D. Kowalski (1996) Nucl. Acids Res. **24:**816-823.

11 Deshpande, A.M., and Newlon, C.S. (1992). The *ARS* consensus sequence is required for chromosomal origin function in *Saccharomyces cerevisiae*. Mol. Cell. Biol. **12:**4305-4313.

12 Zhu, J., Newlon, C.S., and Huberman, J.A. (1992). Localization of a DNA replication origin and termination zone on chromosome *III* of *Saccharomyces cerevisiae*. Mol. Cell. Biol. **12**:4733-4741.

13 Van Houten, J.V., Newlon, C.S. (1990). Mutational analysis of the consensus sequence of a replication origin from yeast chromosome III. Mol. Cell. Biol. **10:**3917-3925.

14 Greenfeder, S.A., and Newlon, C.S. (1992). A replication map of a 61 kb circular derivative of *Saccharomyces cerevisiae* chromosome *III*. Mol. Biol. Cell **3**:999-1013.

15 Theis, J.F., and Newlon, C.S. (1994). Domain B of *ARS307* contains two functional elements and contributes to chromosomal origin function. Mol. Cell. Biol. **14:**7652-7659.

16 Theis, J.F., and Newlon, C.S. (1997). The *ARS309* replicator of *S. cerevisiae* depends upon an exceptional ARS consensus sequence. Proc. Nat. Acad. Sci. USA **94:**10786-10791.
